# Impact of Viral Activators and Epigenetic Regulators on HIV-1 LTRs Containing Naturally Occurring Single Nucleotide Polymorphisms

**DOI:** 10.1155/2015/320642

**Published:** 2015-01-05

**Authors:** Sonia Shah, Vanessa Pirrone, Aikaterini Alexaki, Michael R. Nonnemacher, Brian Wigdahl

**Affiliations:** ^1^Department of Microbiology and Immunology, Drexel University College of Medicine School of Medicine, 245 N. 15th Street, MS1013A, Rm 18301, Philadelphia, PA 19102, USA; ^2^Center for Molecular Virology and Translational Neuroscience, Institute for Molecular Medicine and Infectious Disease, Drexel University College of Medicine School of Medicine, Philadelphia, PA 19102, USA

## Abstract

Following human immunodeficiency virus type 1 (HIV-1) integration into host cell DNA, the viral promoter can become transcriptionally silent in the absence of appropriate signals and factors. HIV-1 gene expression is dependent on regulatory elements contained within the long terminal repeat (LTR) that drive the synthesis of viral RNAs and proteins through interaction with multiple host and viral factors. Previous studies identified single nucleotide polymorphisms (SNPs) within CCAAT/enhancer binding protein (C/EBP) site I and Sp site III (3T, C-to-T change at position 3, and 5T, C-to-T change at position 5 of the binding site, respectively, when compared to the consensus B sequence) that are low affinity binding sites and correlate with more advanced stages of HIV-1 disease. Stably transfected cell lines containing the wild type, 3T, 5T, and 3T5T LTRs were developed utilizing bone marrow progenitor, T, and monocytic cell lines to explore the LTR phenotypes associated with these genotypic changes from an integrated chromatin-based microenvironment. Results suggest that in nonexpressing cell clones LTR-driven gene expression occurs in a SNP-specific manner in response to LTR activation or treatment with trichostatin A treatment, indicating a possible cell type and SNP-specific mechanism behind the epigenetic control of LTR activation.

## 1. Introduction

Over the past decade, targeting the viral entry process, reverse transcriptase, integrase, and protease with highly active antiretroviral therapy (HAART) has prolonged the lives of people infected with HIV-1. However, through various avenues such as cessation of highly active antiretroviral therapy (HAART), the development of drug resistance, and replication of virus in compartments refractile to drug penetration, expansion of HIV-1 viremia or emergence of specific genetic viral variants may rebound from latent reservoirs such as bone marrow progenitor cells, monocytes, and resting memory T cells within the host and repopulate the resident immune and other cellular compartments present in end organs penetrated during the course of HIV disease [1–3]. HIV-1 utilizes cells of the monocyte-macrophage lineage to cross the blood-brain barrier (BBB) and gain entry into the CNS [4–6], thereby promoting HIV-1-associated neuropathogenesis and the development of minor neurocognitive impairment and the severe CNS disease HIV-1-associated dementia (HAD). Perivascular macrophages, located on the parenchymal side of the BBB, likely play a critical role in the pathogenesis of HAD because there is a continuous renewal of the pool through bone marrow-derived macrophages, particularly during systemic and CNS inflammation [6]. In addition, it has recently been shown that infected bone marrow progenitor cells can differentiate into both monocytes and T cells [1], thus potentially serving as a source of HIV-1-infected macrophages and T cells, and they play a critical role in neuroinvasion and progression of CNS disease.

Once viral DNA has integrated into the host genome, it becomes subject to the same epigenetic factors that help to regulate host gene transcription. The formation of nucleosomes and other structures combine and fold together to eventually form a chromosome that compacts and condenses the human genome so that it can be contained within the nucleus. Nucleosomes carry epigenetically inherited information in the form of covalent modifications of their core histones. The nucleosome consists of DNA wrapped around a histone octamer comprised of duplicate copies of the core histones H2A, H2B, H3, and H4, while the H1 histone acts as a linker between nucleosomes. Studies concerning viral transcription have shown that the LTR interacts with nucleosomes Nuc1 and Nuc0 regardless of the integration site. One mechanism through which HIV latency is maintained has been shown to be through the action of histone deacetylases (HDACs) that function to alter the molecular architecture of the HIV-1 LTR and surrounding chromatin. HDACs repress transcription through their ability to covalently modify the lysine tail of core histones through deacetylation, thereby decreasing the access of transcription factors to the DNA. HDACs can be classified into one of three categories designated class I, class II, and class III. Class I HDACs, consisting of HDAC 1, HDAC 2, HDAC 3, and HDAC 8, have been shown to be very effective inducers of virus outgrowth from resting CD4^+^ T cells of aviremic patients [7] compared to class II or class III HDACs. HDAC1 has been shown to be recruited to the LTR by transcription factors such as LSF/YY1, AP-4, NF-*κ*B p50 homodimer, C-myc with Sp1, and CBF-1 by binding near the NF-*κ*B/NFAT binding site [8–12] and has been shown to alter the nucleosome-DNA interaction by making HIV-1 less accessible to host and viral factors. In contrast, histone acetyl transferases (HATs) have been shown to transfer an acetyl group from acetyl-CoA to the lysine tail of histones, thereby leading to greater accessibility of transcription factors and other proteins to the DNA by weakening the DNA-nucleosome interactions. Activated complexes may recruit HATs to the DNA and contribute to the stabilization of the transcriptionally active state of HIV-1 in either a Tat-independent or Tat-dependent manner [13]. The NF-*κ*B p50-RelA heterodimer and Sp1 interact with the HAT p300, required for maximal Tat activity, helping to enhance recruitment to the LTR to optimize transcription of viral DNA [14–18].

Genetic variation within the HIV-1 genome naturally occurs because of the low fidelity of reverse transcriptase, coupled with the selective pressures brought about within the host such as HAART, recreational drug use, immunological pressures, viral recombinatory events, host cell phenotype, and rates of virus production [19, 20]. These events lead to the development and expansion of single nucleotide polymorphisms (SNPs) throughout the genome, including the LTR, within sites where host transcription factors and viral regulatory proteins bind, altering the way in which the LTR drives viral transcription and overall viral gene expression. The resultant viral quasispecies that develop are likely formed and maintained in a variety of cellular and tissue niches that ultimately maintain specific sets of quasispecies to form viral reservoirs in susceptible cell types and end organ tissues based on these selective pressures [21–28].

Activating and repressing host transcription factors and viral proteins are ultimately recruited to transcription factor binding sites and the transcription complex within different cell populations, subsequently altering the basal and inducible rates of viral gene expression based on the quantitative and qualitative availability of specific factors within different cellular compartments at any given time [13, 29–32]. The differential regulation of the LTR potentially impacts viral replication in both a cell type-, and tissue-specific manner [25, 33–35] that may impact the course of HIV-1 disease [36]. Sequence variation within the HIV-1 LTR can result in functional alterations within cis-acting transcription factor binding sites in the LTR during the evolution of quasispecies, resulting in altered promoter activity [21, 25, 28] and transcription factor binding [23, 24, 26, 27]. Specific nucleotide changes within the LTR, such as a 3T variation in C/EBP site I or a 5T variation within the Sp III binding site, abrogate the binding of cognate transcription factors to their corresponding binding site and may correlate more the severe stages of HIV-1 disease [23, 24, 26, 27, 37–39]. Additionally, the 5T SNP occurs almost as frequently as the consensus sequence for Sp site III in the Drexel Medicine CNS AIDS Research and Eradication Study (CARES) (data not shown).

Previous studies have shown that populations of cells that were stably transfected with the parental LAI LTR and its sequence variants 3T within C/EBP site I and 5T within Sp site III exhibited altered phenotypic properties of the binding site and LTR when placed within the context of different LTR backbones and under a number of different stimulatory conditions within the context of stably integrated gene expression assays [40]. Consequently, in order to better understand the mechanisms of the cellular phenotypes observed in the sequence variants with respect to LTR activity and viral replication within the context of a homogeneous chromatin-based microenvironment, clonal cell populations were developed from the previous populations of stably transfected TF-1, U-937, and Jurkat cells.

## 2. Material and Methods

### 2.1. Cell Culture

The TF-1 CD34^+^ erythromyeloid leukemia cell line (ATCC, Manassas, VA) was grown in RPMI 1640 medium with L-glutamine (Cellgro, Herndon, VA) supplemented with 10% heat-inactivated fetal bovine serum (FBS; GemCell, Sacramento, CA), antibiotics (penicillin and streptomycin, at a concentration of 0.04 mg/mL each, Cellgro), glucose (4.5 g/mL, Cellgro), sodium pyruvate (1 mM, Cellgro) and HEPES (10 mM, Cellgro), and recombinant human granulocyte-macrophage-colony stimulating factor (rhGM-CSF, 2 ng/mL; eBioscience, San Diego, CA). The cells were maintained at a density between 1 and 5 × 10^5^ cells/mL.

The U-937 human promonocytic cell line (ATCC, CRL-1593.2) was grown in RPMI 1640 medium with L-glutamine supplemented with 10% heat-inactivated FBS, antibiotics (penicillin and streptomycin, at a concentration of 0.04 mg/mL each), glucose (4.5 g/mL), sodium pyruvate (1 mM), and HEPES (10 mM). The cells were maintained at a density between 1 and 5 × 10^5^ cells/mL.

The human T-cell line, Jurkat (ATCC, TIB-152), was grown in RPMI-1640 media with L-glutamine supplemented with 10% heat-inactivated FBS, sodium bicarbonate (0.05%, Cellgro, Herndon, VA), and antibiotics (penicillin, streptomycin, and kanamycin at 0.04 mg/mL each, Cellgro, Herndon, VA). The cells were maintained at a density between 1 × 10^5^ and 1 × 10^6^ cells/mL. All cells were maintained at 37°C in 5% CO_2_ at 90% relative humidity.

### 2.2. Plasmid Construction and Site-Directed Mutagenesis

The HIV-1 LAI LTR sequence was amplified by polymerase chain reaction (PCR) from previously published LAI-LTR-Luc reporter constructs [41, 42]. The LAI LTR wild type, 3T C/EBP site I, 5T Sp site III, and 3T5T double variant were all constructed into the pEGFP-N1 vector (Clontech, Mountain View, CA) as previously described [40].

### 2.3. Stable Transfection of TF-1, U-937, and Jurkat Cells and Stably Transfected Cell Clone Development

TF-1, U-937, and Jurkat cells were transfected with the abovementioned constructs using the AMAXA nucleofector system and AMAXA kit V (Lonza). Transfected cells were passaged under 800 ng/mL G418 (Mediatech) selection beginning 24 hours after transfection and passaged under G418 selection for at least 2 months to develop stably transfected populations. These cell populations were each serially diluted to a final concentration of 1 cell/mL media and plated into 96-well plates, where they were marked and tracked for growth. Once each clonal cell became a confluent clonal population within the well, they were moved to a larger well with more media until they could be transferred to flasks and propagated normally. Basal levels of LTR-driven GFP expression within each cell population and clonal population were measured using flow cytometry. The construction and grouping of these clones are further described in [40]. Because a different number of clones were generated for each LTR (wt, 3T, 5T, and 3T5T) in each of the different cell lines (TF-1, U937, and Jurkat), three representative clones, which typify all of the clones, were selected for further analysis as shown in the figures.

### 2.4. Assessment of GFP Expression Utilizing Flow Cytometry

TF-1, U-937, and Jurkat clonal cell lines were washed with FCM buffer (Hanks balanced saline solution (HBSS) (Mediatech, Herndon, VA), 3% FBS, and 0.02% NaN_3_) and aliquots of 1 × 10^6^ cells were fixed with paraformaldehyde (1%). FCM analysis was performed utilizing a Calibur flow cytometer (BD Biosciences) with 100,000 events collected for each analysis, and the results were analyzed using Flowjo version 6.1.1 software (Tree Star, San Carlos, CA). All experiments were completed in triplicate in three independent experiments. Representative histograms are displayed within each figure.

### 2.5. Cytokine Stimulation, Histone Deacetylase Inhibitor Treatment, and Tat Transfection of Cells

Stably transfected cells were exposed to human recombinant TNF-*α* (e-Biosciences, San Jose, CA) at a concentration of 20, 50, 100, 200, or 300 ng/mL. Cells were exposed to cytokine for 24 hours, washed, and subsequently harvested for determination of HIV-1 LTR activity as described above. Separately, stably transfected cell lines were transiently transfected with Tat101 (300 ng) using the Amaxa nucleofector system and Ingenio electroporation solution (Mirus Bio) and harvested after 24 hours. Within the context of Tat, untreated refers to transfection with the parental pcDNA3.1 plasmid without the Tat gene (in other words, empty vector). Independently, cells were also exposed to the HDAC inhibitor trichostatin A (TSA) (400 nM) for 24 hours with or without TNF-*α* stimulation as indicated above and then LTR-driven GFP transcription was determined using flow cytometry as described above.

## 3. Results

### 3.1. Non-GFP-Expressing Stably Transfected TF-1 Cell Clone Lines Containing the 3T5T-LTR Can Be Induced to Drive GFP Expression with TNF-*α* Stimulation

**Figure 1:**
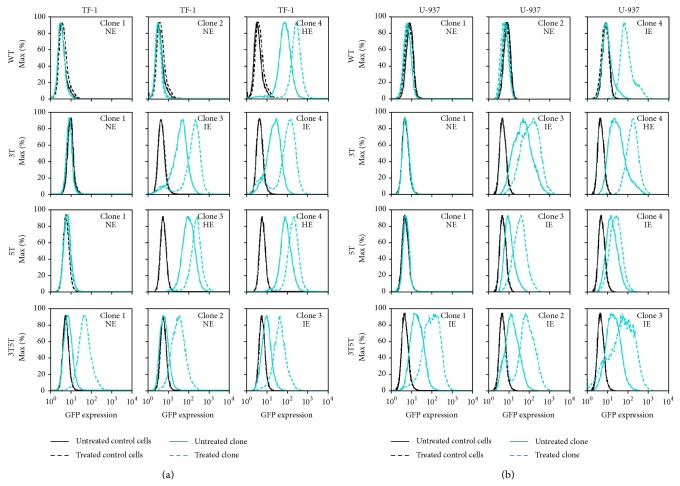
TNF-α induction of LTR-driven GFP expression is dependent on the basal LTR expression phenotype in hematopoietic progenitor TF-1 cell clones as well as promonocytic U-937 cell clones. Both TF-1 and U-937 cells stably transfected with the LAI LTR (WT, 3T, 5T, and 3T5T) were serially diluted in order to obtain 1 cell in 1 mL of media (approximately one cell in 10 wells of a 96-well plate). Cell clone populations were propagated from the single cell and then were analyzed using flow cytometry for their basal GFP expression. The clonal populations were then designated in one of three categories, nonexpresser (NE), intermediate expresser (IE), and high-expresser (HE), based on their geometric mean fluorescence intensity (MFI) and their percent cell positive values. Each individual cellular clone across all four backbones was treated with TNF-α (20 ng/mL). All experiments were completed in triplicate in three independent experiments. Representative histograms showing levels of GFP expression obtained with the untreated stably transfected cell clone (solid turquoise line) compared to the levels of GFP expression obtained with treated stably transfected cell clone (dashed turquoise line), treated WT TF-1 and U-937 cells (dashed black line), and untreated WT TF-1 and U-937 cells (solid black line) are shown. (a) The NE LAI WT, 3T, and 5T LTR-containing clones could not be induced into driving GFP expression, whereas their GFP-expressing clone counterparts could be induced. All the NE and the IE 3T5T LTR-containing TF-1 clones could be induced to express higher levels of GFP expression. (b) The NE LAI WT, 3T, and 5T LTR-containing clones could not be induced into driving GFP expression, whereas their GFP-expressing clone counterparts could be induced. All of the IE 3T5T LTR-containing clones could be induced to express high levels of GFP.

It was of interest to determine if stimulation of the non-GFP-expressing (NE) and low/intermediate-expressing (IE) LTRs with inflammatory cytokines could induce the LTRs to drive GFP expression because they may be able to represent cell models for “promoter latency and reactivation” within the bone marrow progenitor cell type with LTRs containing naturally occurring SNPs. To effectively study how the LTR functions to drive GFP expression with TNF-*α* stimulation from the standpoint of a more homogeneous cell, cell clones were developed from the TF-1 stably transfected cell lines. Each cell clone population was treated with TNF-*α* (20, 50, 100, 200, or 300 ng/mL) for 24 hours and then the levels of LTR-driven GFP expression were assessed by flow cytometry. Each concentration of TNF-*α* resulted in the same level of LTR-driven GFP expression (data not shown); therefore, each panel within Figure 1(a) represents a separate cell clone treated with TNF-*α* (20 ng/mL). HE-GFP LTRs are induced much more efficiently with the introduction of cytokines and as such were used as a control to show that the LTR was in fact able to be induced.

NE cell populations within the WT, 3T, and 5T LTR stably transfected TF-1 cell clones could not be stimulated by TNF-*α* to drive GFP expression (Figure 1(a)). All of the IE as well as the high-expressing (HE) WT, 3T, and 5T LTR-containing stably transfected TF-1 cell clones were stimulated to higher levels of GFP transcription (Figure 1(a)). Treatment of cell clones containing the NE and IE 3T5T LTR resulted in activation of the LTR and LTR-driven GFP expression to HE levels (Figure 1(a)). Cell clones containing the 3T or 3T5T LTRs had the greatest activation potential when compared to the other cell clones.

### 3.2. U-937 Clones Containing Non-GFP-Expressing LTR Phenotypes Cannot Be Induced by TNF-*α* into Driving GFP Expression

To determine if the NE and IE phenotypes observed within the U-937 cell clones could be induced into expression, each of the cell clones was treated with the various concentrations of TNF-*α* mentioned previously. Interestingly, note that the 3T5T double variant only resulted in IE clones in U-937 cells as previously described [40]. Each concentration of TNF-*α* resulted in the same levels of LTR-driven GFP expression (data not shown); therefore, each panel within Figure 1(b) represents separate cell clones treated with TNF-*α* (20 ng/mL).

With TNF-*α* stimulation, the HE cell clone containing the LAI 3T LTR drove a higher level of GFP expression; however, there was only a small change when compared to LTR-driven GFP expression obtained under basal conditions (Figure 1(b)). In contrast, under the same conditions, the NE cell clones containing the LAI WT, 3T, and 5T LTRs were not stimulated to drive GFP expression. As seen in the TF-1 cell clones (Figure 1(a)), IE U-937 cell clones were able to be induced and drive higher levels of GFP expression (Figure 1(b)). Interestingly, the IE phenotype within the U-937 LAI WT cell clone exhibited the largest difference in unstimulated versus stimulated MFI (33.4 and 80.7, resp.) and percent positive GFP-expressing cell number (9.67% and 98.5%, resp.) when comparing unstimulated against stimulated GFP-expressing clones within all the U-937 cell clones. The clones containing the 3T or 3T5T LTRs had the largest activation potential when compared to basal activity as was seen in the stably transfected TF-1 cells (Figure 1(a)).

### 3.3. IL-1*β* Stimulation of NE 3T5T LTR Containing Hematopoietic Progenitor Cell Clone Lines Does Lead to LTR Induced GFP Expression

**Figure 2:**
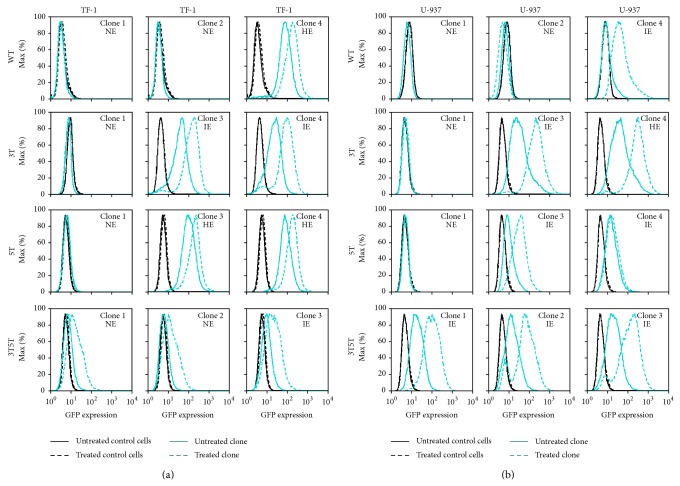
IL-1β induction of HIV-1 transcription is dependent on basal LTR expression phenotype in TF-1 and U-937 cell clones. TF-1 and U-937 stably transfected cell clones containing the WT, 3T, 5T, or 3T5T LAI LTRs were treated with IL-1β (20 ng/mL) for 24 hours and then analyzed using flow cytometry for their stimulated GFP expression. All experiments were completed in triplicate in three independent experiments. Representative histograms showing levels of GFP expression obtained with the untreated stably transfected cell clone (solid turquoise line), the treated stably transfected cell clone (dashed turquoise line), untreated WT TF-1 and U-937 cells (solid black line), and treated WT TF-1 and U-937 cells (dashed black line) are shown. Designations of nonexpressing (NE), intermediate-expressing (IE), and high-expressing (HE) are determined based on basal levels of LTR-driven gene expression within each clone. (a) LTRs from NE WT, 3T, and 5T TF-1 cells were unable to be induced into driving GFP expression, whereas the cells containing NE 3T5T LTRs were activated following treatment. Additionally, active LTRs could be induced to drive higher levels of GFP expression. (b) LTRs from NE U-937 cells were unable to be induced into driving GFP expression, whereas the cells containing active LTRs could be induced to drive higher levels of GFP expression.

To continue analyzing the effects of proinflammatory cytokines on LTR activity within LTRs containing SNPs of interest, the TF-1 stably transfected cell clones were treated with IL-1*β* (20, 50, 100, 200, or 300 ng/mL) for 24 hours and then the levels of LTR-driven GFP expression were assessed by flow cytometry. Each concentration of IL-1*β* resulted in similar levels of LTR-driven GFP expression (data not shown); therefore, each panel within Figure 2(a) represents a separate cell clone treated with IL-1*β* (20 ng/mL). HE-GFP LTRs are induced much more efficiently with the introduction of cytokines and as such were used as a control to show that the LTR was in fact able to be induced. Non-GFP-expressing cell clones within the WT, 3T, and 5T LTR stably transfected cell clones could not be stimulated by IL-1*β* to drive GFP expression (Figure 2(a)). The IE 3T LTR-containing and HE WT and 5T LTR-containing cell clones were stimulated, from basal to higher levels of GFP transcription with IL-1*β* stimulation (Figure 2(a)). The NE and the IE clones containing the 3T5T LTR could be induced to drive intermediate levels of GFP expression (Figure 2(a)). Cell clones containing the 3T or 3T5T LTRs had the largest increase in LTR-driven gene expression when compared to basal activity.

### 3.4. U-937 Clones Containing Nonexpressing LTR Phenotypes Cannot Be Induced by IL-1*β* into Driving GFP Expression

To determine if the NE and IE phenotypes observed within the U-937 cell clones could be induced into expression, each of the cell clones was treated with IL-1*β* for 24 hours. IL-1*β* stimulation of the expressing cell clones containing the LAI WT, 3T, 5T, and 3T5T LTRs drove higher levels of GFP expression; however, one 5T LTR-containing GFP-expressing clone (clone 4) was not stimulated into higher expression. In contrast, under the same conditions, the NE clones containing the LAI WT, 3T, and 5T LTRs were not stimulated to drive GFP expression (Figure 2(b)). Cell clones containing the 3T or 3T5T LTRs exhibited the largest increases in LTR-driven gene expression when compared to basal activity.

### 3.5. Tat101 Transactivation of Non-GFP-Expressing Stably Transfected TF-1 Cell Clones Does Not Lead to LTR-Induced GFP Expression

The full-length Tat is comprised of 101 amino acids and is coded from 2 exons. Tat101 is the naturally occurring Tat within the HIV-1 LAI strain of virus. Tat101 has been shown to be able to activate nuclear translocation of NF-*κ*B [43–46] and can greatly increase viral replication through interaction with the transactivation response (TAR) element and subsequent recruitment of host transcription factors and viral proteins to the LTR. To observe the effects of Tat101 on variant LTR-driven gene transcription and overall expression within a single cell, stably transfected TF-1 cell clones were transactivated with Tat101 (300 ng) through transient transfection with a pcDNA3.1 plasmid containing the Tat101 gene. LTR-driven gene expression was subsequently analyzed using flow cytometry.

**Figure 3:**
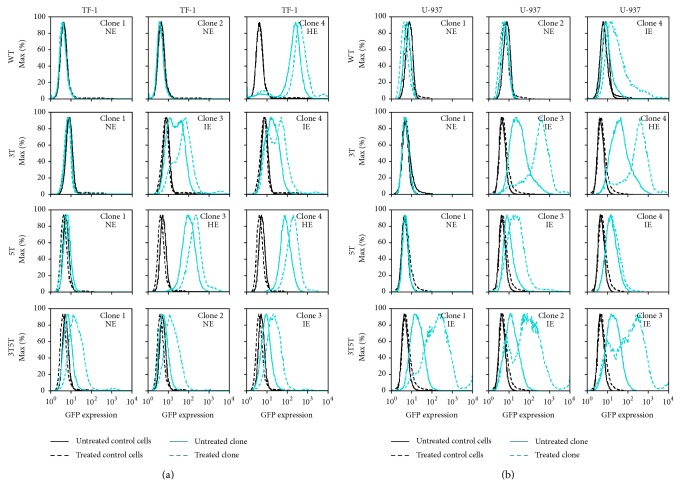
Tat101 transactivation leads to increases in LTR-driven gene transcription within GFP-expressing TF-1 and U-937 cell clones. TF-1 and U-937 stably transfected cell clones containing the WT, 3T, 5T, or 3T5T LAI LTRs were transfected with Tat101 (300 ng) using the Ingenio electroporation solution with an Amaxa nucleofector device and were analyzed for GFP expression using flow cytometry 24 hours after transfection. All experiments were completed in triplicate in three independent experiments. Representative histograms show GFP expression levels from the untreated stably transfected cell clone (solid turquoise line), the treated stably transfected cell clone (dashed turquoise line), untreated WT TF-1 and U-937 cells (solid black line), and treated WT TF-1 and U-937 cells (dashed black line). Within the context of Tat, untreated refers to transfection with the parental pcDNA3.1 plasmid without the Tat gene (in other words, empty vector). Designations of nonexpressing (NE), intermediate-expressing (IE), and high-expressing (HE) are determined based on basal levels of LTR-driven gene expression within each clone. (a) LTRs from NE WT, 3T, and 5T TF-1 cells were unable to be induced into driving GFP expression, whereas LTRs from NE 3T5T TF-1 cells could be induced to drive higher levels of GFP expression. Additionally, the cells containing active LTRs could be induced to drive higher levels of GFP expression. (b) LTRs from NE cells were unable to be induced into driving GFP expression, whereas the cells containing active LTRs could be induced to drive higher levels of GFP expression.

The LTRs from NE TF-1 cell clones containing the WT, 3T, and 5T LTRs could not be transactivated by Tat101 to drive GFP expression (Figure 3(a)). Both IE and HE cell clones containing the WT, 3T, or 5T LTRs were able to be stimulated resulting in an increase in LTR-driven GFP expression compared to basal activity (Figure 3(a)). In addition, Tat101 transactivation of the NE, as well as the IE, cell clones containing the 3T5T LTR resulted in intermediate levels of GFP expression. Clones containing the 3T5T LTRs had the largest difference in LTR-driven GFP expression when compared to basal activity (Figure 3(a)).

### 3.6. Tat101 Transactivation of U-937 Clones with Non-GFP-Expression Phenotype Does Not Induce LTR-Driven GFP Expression

To determine if full-length Tat101 transient transfection of the NE, IE, and HE GFP-expressing phenotypes observed within the U-937 cell clones could transactivate each of the LTRs containing SNPs of interest, each of the cell clones was transiently transfected with 300 ng of pcDNA3.1 plasmid containing Tat101 gene. Tat101 transactivation of the IE and HE cell clones containing the LAI WT, 3T, 5T, and 3T5T LTRs drove higher levels of GFP expression with the largest changes observable within the 3T and 3T5T intermediate expressing cell clones when compared to basal GFP expression (Figure 3(b)). Under the same conditions, the NE clones containing the LAI WT, 3T, and 5T LTRs, as well as IE 5T LTR cell clone 4, were not transactivated and did not respond to Tat101 transactivation (Figure 3(b)).

### 3.7. Treatment of Stably Transfected TF1 and U-937 Cells with TSA and TNF-*α* Results in Little to No LTR-Driven Gene Expression in GFP-Expressing Clones Containing HIV-1 LAI LTRs

Trichostatin A (TSA) is a general HDAC inhibitor that functions by acetylating lysine residues on class I and II histone tails of nucleosomes, thereby allowing for the integrated HIV-1 LTR DNA to become more accessible to host transcription factors and polymerases. Previous studies that have utilized TSA as an HDAC inhibitor to activate HIV-1 expression generally use selected concentrations ranging from 0 to 1.5 *μ*M across many different cell types cultured in vitro. To study the effect of the general HDAC inhibitor TSA on NE and IE GFP-expressing stably transfected cell clones, both TF-1 and U-937 stably transfected cell clones across LTRs containing the SNPs of interest (3T, 5T, and 3T/5T) were treated with 100 nM TSA for 24 hours and then were analyzed for stimulated GFP expression. Interestingly, TSA treatment of most cell clones resulted in no change in LTR-driven gene expression whether they were NE, IE, or HE (data not shown). Stably transfected TF-1 and U-937 cell clones were also treated with the specific HDAC 1 inhibitor valproic acid (VPA) at 0.5, 1, and 1.5 mM in the absence or presence of TNF-*α* and showed no effect on LTR-driven gene expression (data not shown), indicating that one of the other HDACs, or possibly a combination of HDACs, may play a role in histone suppression of the LTR.

**Figure 4:**
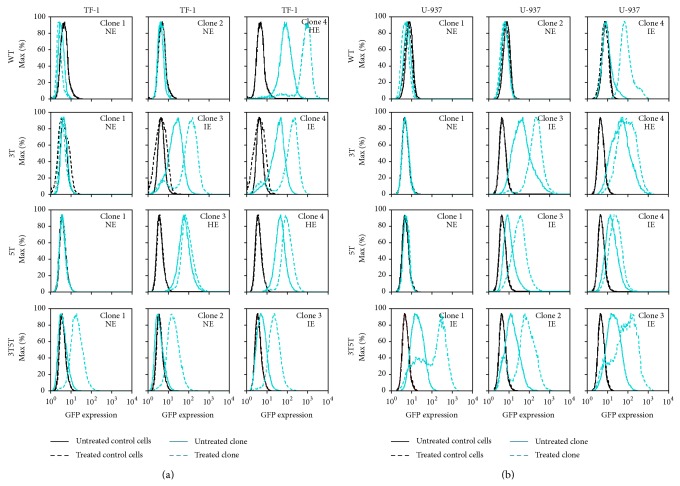
HIV-1 LAI non-GFP-expressing LTR is not inducible by the combination of TSA and TNF-α above levels seen with TNF-α alone in stably transfected TF-1 and U-937 cell clones. Stably transfected TF-1 cell clones containing the WT, 3T, 5T, or 3T5T LAI LTRs were treated with 100 nM trichostatin A (TSA) in combination with TNF-α (20 ng/mL) for 24 hours, along with untreated clone controls as well as untreated and treated TF-1 cells (a) and U-937 cells (b). After 24 hours, cells were analyzed for GFP expression using flow cytometry. All experiments were completed in triplicate in three independent experiments. Representative histograms show levels of GFP expression of the untreated cells (solid black line) compared to the treated cells (dashed black line), and untreated stably transfected cell clones (solid turquoise line) compared to the treated cell clones (dashed turquoise line). TSA alone did not result in any alterations in gene expression within any of the cell clones examined.

When TSA acetylates the lysine residues of histone tails, integrated DNA may not have the necessary transcription factors around it in abundance to be able bind to available promoter binding sites, despite the fact that the DNA is now presumably accessible to those host and viral factors. Therefore, it was hypothesized that treatment of cells with a combination of TSA and the proinflammatory cytokine TNF-*α* would potentially increase the presence of host transcription factors, particularly NF-*κ*B, at the LTR. To observe if the combination treatment of TSA with a stimulatory proinflammatory cytokine could result in increases in LTR-driven gene expression of NE stably transfected TF-1 and U-937 cells, the cell clones were treated with TSA (100 nM) in combination with TNF-*α* (20 ng/mL) for 24 hours. After treatment, GFP expression was analyzed using flow cytometry. TSA treatment with varying concentrations of TNF-*α* (20, 50, 100, and 200 ng/mL) all resulted in the same level of LTR-driven gene expression in both cell types (data not shown); therefore, the final TNF-*α* concentration of 20 ng/mL was utilized in these studies. In both TF-1 cells (Figure 4(a)) and U-937 cells (Figure 4(b)), the level of LTR-driven gene expression is similar to that observed with TNF-*α* treatment alone. This implies that TSA does not increase gene expression either alone or in combination with TNF-*α* in either cell type.

### 3.8. Non-GFP-Expressing Jurkat Clone Containing the 5T LTR Can Be Induced to Drive GFP Expression under TNF-*α* Stimulation

After determining the effects of LTR driven gene expression within cells of the hematopoietic and monocytic lines, it was of interest to determine if LTR activation by inflammatory cytokines could also induce the stably transfected Jurkat cell clones containing the WT, 3T, and 5T LTRs into driving GFP expression within quiescent T cells with LTRs containing naturally occurring SNPs and to determine whether under selected conditions some cells that seem unresponsive to LTR activators could become activated under specific intracellular conditions. The Jurkat cell clones were treated with a range of TNF-*α* concentrations to determine whether inflammatory cytokine induction of the LTR could drive GFP expression in these cells. As observed within the TF-1 and U-937 cell clones, titration of TNF-*α* concentrations resulted in very similar levels of LTR-driven GFP expression, and therefore, for the purposes of this figure and subsequent experiments, only the 20 ng/mL concentration of TNF-*α* was used.

**Figure 5:**
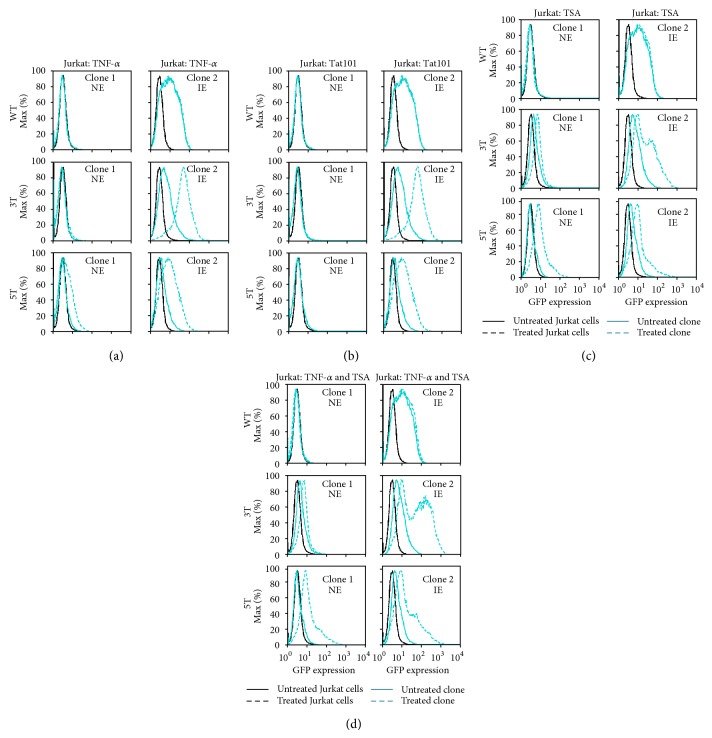
LTR-driven gene expression is induced in non-GFP-expressing LAI LTR clones stimulated with various treatments in T-cell clones. Jurkat cells stably transfected with the LAI LTR (WT, 3T, and 5T) were serially diluted in order to obtain one cell in 1 mL of media (approximately 1 cell in 10 wells of a 96-well plate. Cell clone populations were propagated from the single cell stage and then were analyzed using flow cytometry for their basal GFP expression. The Jurkat clonal cell populations were then designated in one of three categories: nonexpresser (NE), intermediate-expresser (IE), and high expresser (HE), based on their geometric mean fluorescence intensity (MFI) and their percent cell positive values. (a) Jurkat cell clones containing all three backbones were treated with TNF-α (20 ng/mL). Levels of GFP expression obtained with the untreated stably transfected cell clone (solid turquoise line) compared to the levels of GFP expression obtained with the treated stably transfected Jurkat cell clone (dashed turquoise line), treated WT Jurkat cells (dashed black line), and untreated WT Jurkat cells (solid black line) are shown. The NE and IE LAI WT Jurkat clones could not be induced into expression. The 3T NE clone could not be induced to drive GFP expression, whereas the IE clone was induced. The NE and IE clones containing the 5T LTR could both be induced into driving GFP expression when stimulated with TNF-α. (b) Jurkat stably transfected cell clones containing the WT, 3T, or 5T LAI LTRs were transfected with 300 ng Tat101 using the Ingenio electroporation solution with an Amaxa nucleofector device and were subsequently analyzed for GFP expression using flow cytometry after 24 hours. Within the context of Tat, untreated refers to transfection with the parental pcDNA3.1 plasmid without the Tat gene (in other words, empty vector). The NE and IE WT LTR and the NE 3T and 5T LTR containing clones could not be transactivated following Tat101 transfection. Expressing clones containing the 3T and 5T LTRs were transactivated to drive higher levels of GFP expression. (c) Stably transfected Jurkat cell clones containing the LAI WT, LAI 3T, or LAI 5T LTRs were treated TSA (400 nM) for 24 hours, along with untreated clone controls as well as untreated and treated Jurkat cells. After 24 hours, cells were analyzed for GFP expression using flow cytometry. The NE 3T and 5T LTR cell clones could be induced into expression with TSA treatment, whereas neither the NE or IE WT cell clones could be induced. (d) Stably transfected Jurkat cell clones containing the LAI WT, LAI 3T, or LAI 5T LTRs were treated with TNF-α (20 ng/mL) and TSA (400 nM) for 24 hours, along with wild type Jurkat cells. After 24 hours, cells were analyzed for GFP expression using flow cytometry. TSA with TNF-α treatment resulted in a small increase in the NE 5T LAI LTR-containing cell clone compared to TSA treatment alone. The combination treatment did not lead to increases in 3T LAI LTR-driven GFP expression within the NE 3T LAI LTR cell clone. (a–d) All experiments were completed in triplicate in three independent experiments. Representative histograms showing levels of GFP expression obtained with the untreated stably transfected cell clone (solid turquoise line), the treated stably transfected cell clone (dashed turquoise line), untreated WT Jurkat cells (solid black line), and treated WT Jurkat cells (dashed black line) are shown.

As expected, based on previous results with other cell lines, the NE WT and 3T cell clones, when stimulated, were not able to be induced to drive any level of GFP expression (Figure 5(a)). Interestingly, under stimulated conditions, the IE clones containing the LAI WT LTR remained completely unchanged by TNF-*α* activation (Figure 5(a)). The clonal cells containing the IE 3T LAI LTR and 5T LAI LTR were able to be stimulated with TNF-*α* to drive higher levels of GFP expression. Unexpectedly, the NE 5T LAI LTR could be stimulated by TNF-*α* to drive intermediate levels of GFP expression (Figure 5(a)). This result is important because it suggests that reactivation from NE cells could be either cell type- or SNP-specific and that the location of the LTR within the genome, in the context of epigenetic control and integration, could play a role in the ability of the LTR to be expressed.

### 3.9. Tat101 Does Not Transactivate the NE WT, 3T, or 5T LTRs in Jurkat Cells

To determine if the NE phenotypes observed within the WT, 3T, and 5T LAI LTR-containing Jurkat cell clones could be transactivated, each of the cell clones was transiently transfected with 300 ng of a pcDNA3.1 plasmid containing the Tat101 gene and then the levels of LTR-driven GFP expression were assessed by flow cytometry 24 hours after transient transfection. Each panel shown contains a representative histogram of one of three transient transfections of each individual clone. Tat101 transactivation of the IE cell clones containing the LAI 3T and 5T LTRs drove higher levels of GFP expression when compared to basal expression (Figure 5(b)). In contrast, under the same conditions, the NE clones containing the LAI 3T and 5T LTRs were not transactivated and did not drive GFP expression (Figure 5(b)). Tat 2w within either the NE or IE LAI WT LTR containing cell clones (Figure 5(b)).

### 3.10. Treatment of Stably Transfected Jurkat Cells with TSA Stimulates LTR-Driven Gene Expression in Clones Stably Transfected with the 3T and 5T LAI LTRs

Previous studies have used a concentration of 400 nM TSA to treat latent HIV-1-infected Jurkat T cells [13, 47, 48] to stimulate LTR-driven gene transcription. To study the effect of the general HDAC inhibitor TSA on NE and IE GFP-expressing stably transfected Jurkat cell clones containing the WT, 3T, and 5T LAI LTRs, they were treated with TSA (400 nM) for 24 hours. TSA-stimulated LTR-driven gene expression was analyzed after treatment using flow cytometry.

TSA treatment did not alter LTR-driven gene expression within either the NE or IE cell clones containing the WT LAI LTR (Figure 5(c)). In contrast, the intermediate GFP-expressing cell clones containing the 3T and 5T LAI LTRs were stimulated to express higher levels of GFP expression (71.7% and 53.2%, resp.) compared to basal levels (32.6% and 16.5%) (Figure 5(c)). Interestingly, the non-GFP-expressing clones stably transfected with the 3T and 5T LAI LTRs were stimulated to express higher levels of GFP expression (23.5% and 43.1%, resp.) when compared to basal levels (11.2% and 5.05%, resp.) (Figure 5(c)).

### 3.11. Treatment of Non-GFP-Expressing Stably Transfected Jurkat Cells with TSA and TNF-*α* Stimulates LTR-Driven Gene Expression in Clones Containing the 5T LAI LTR above That Observed with TSA Alone

TSA treatment of TF-1 and U-937 cell clones did not result in any increase in LTR-driven gene expression above basal levels (as described above). The treatment of cells with a combination of TSA and the proinflammatory cytokine TNF-*α* was hypothesized to potentially increase the presence of host transcription factors, particularly NF-*κ*B, at the LTR, thereby increasing the level of LTR-driven gene activation. However, in TF-1 and U-937 cells, activation following combination treatment did not exceed levels observed following treatment with TNF-*α* alone. However, TSA treatment of NE and IE 3T and 5T Jurkat cell clones did result in higher expression levels above basal activity. Therefore, to observe if the combination treatment of TSA with a stimulatory proinflammatory cytokine could result in increases in LTR-driven gene expression of NE stably transfected Jurkat cells, the cell clones were treated with TSA (400 nM) in combination with TNF-*α* for 24 hours. After treatment, GFP expression was analyzed using flow cytometry. TSA treatment with varying concentrations of TNF-*α* (20, 50, 100, and 200 ng/mL) all resulted in the same level of LTR-driven gene expression (data not shown); therefore, the final TNF-*α* concentration of 20 ng/mL was utilized in these studies.

Combination treatment of TSA and TNF-*α* of the NE and IE Jurkat clones containing the WT LAI LTR did not result in an increase of GFP expression (Figure 5(d)). As expected, when the 3T LAI LTR- and 5T LAI LTR-containing IE clones were treated with the combination of TSA and TNF-*α*, the LTRs were stimulated to drive higher levels of GFP expression (Figure 5(d)). With the combination treatment, the 3T LTR within the NE Jurkat clone was stimulated to increase GFP expression at the same level as TSA treatment alone (Figures 5(c) and 5(d)), indicating that, with this clone, the addition of TNF-*α* did not intensify the effects of TSA. In contrast, combination treatment with TSA and TNF-*α* of the NE Jurkat clone containing the stably transfected 5T LAI LTR led to intermediate levels of GFP expression compared to basal levels and higher levels of LTR-driven GFP expression compared to TSA treatment alone (Figure 5(d)).

## 4. Discussion

We have previously demonstrated and identified specific single nucleotide polymorphisms (SNPs) within C/EBP site I (3T) and Sp site III (5T) that correlate with increased HIV-1 disease severity, HAD, as well as altered transcription factor binding to those sites [23, 24, 26, 37, 38]. Additionally, studies have shown that sequence variation within the LTR can result in functional alterations within cis-acting transcription factor binding sites during the evolution of quasispecies, resulting in altered promoter activity [21, 25, 28], altered transcription factor binding [23, 24, 26], and LTR-driven gene expression [13]. In this study, the effects of the 3T, 5T, and 3T5T SNPs on LTR function and how these SNPs contribute to the regulation of viral transcription and potentially facilitate the maintenance of HIV-1 latency and viral reactivation within hematopoietic progenitor cells (HPCs), monocytes, and CD4+ T cells were explored.

Each of the cell clones was treated with the proinflammatory cytokines TNF-*α* and IL-1*β* and was transiently transfected with Tat101. Within NE TF-1 and U-937 clones containing the WT, 3T, or 5T LTRs under investigation, and neither stimulation nor Tat-transactivation led to any increases in LTR-driven gene expression indicating that perhaps there are epigenetic regulators or differences in integration site that may control latency within these cells, as has been shown in other studies [29, 47, 49–51] (Figures 1–4). In contrast, stimulation of the three 3T5T TF-1 cell clones resulted in activation of the LTR and intermediate levels of LTR-driven GFP expression (Figures 1 and 2).

Following both TNF-*α* and IL-1*β* stimulation, in TF-1 as well as U-937 cells, the greatest activation potential was seen in the clones containing the 3T or 3T5T LTRs (Figures 1 and 2). TNF-*α* stimulation of the IE Jurkat clones containing the 3T or 5T LAI LTRs resulted in increased LTR-driven gene expression. The lack of stimulation observed in the IE Jurkat clone containing the WT LAI LTR could be due to the location within the genome that the LTR has integrated and/or other epigenetic controls preventing further LTR-driven gene expression [52–56]. Interestingly, TNF-*α* stimulation led to induction of LTR-driven gene expression from the NE Jurkat clone containing the 5T LAI LTR (Figure 5(a)), which could be a result of the gene's availability to utilize host transcription factors under activated conditions. The robust nature of cytokine-mediated induction of 3T and 3T5T LTR-induced gene expression within IE TF-1 cell clones, the 3T, 5T, and 3T5T LTRs-induced gene expression within IE U-937 cell clones, and the 5T LTR-induced gene expression within the Jurkat cell clone suggest that there is a SNP-specific and cell-specific mechanisms of action that lead to alterations in LTR-driven gene expression.

Tat has been shown to activate the LTR through nuclear translocation of NF-*κ*B [43–46] as well as through its interaction with the TAR element and recruitment of histone acetyl transferases, host and viral factors, pTEFb, and RNA polymerase to the LTR [32]. Tat transactivation of the LTRs within the stably transfected cell clones resulted in some differences in LTR-driven gene expression possibly through some of the different ways in which cytokines, transcription factors, chromatin modifying proteins, and Tat interact with the LTR [35, 57]. It appears that specific genotypes, such as the 3T or the 3T5T LTR, result in enhanced LTR responsiveness to Tat transactivation within HPCs or monocytes. The 3T and 5T LTRs within Jurkat cell clones also resulted in large change in LTR-driven gene expression within those cells; however, since we have not yet been able to obtain Jurkat cell clones containing the 3T5T LTR, we have not been able to compare the differences in Tat transactivation between the three cell types containing the same LAI LTR. It is possible that the less robust inductions obtained with LTR-driven gene expression resulting from Tat and proinflammatory cytokine exposure of the TF-1, U-937, and Jurkat cell clones containing the 5T LAI LTR could help it to maintain presence within the host when placed within the context of the replication competent virus and not be as quickly eliminated by the immune system or by HAART as the virus containing the 3T or 3T5T LTRs when placed in stimulated conditions.

TF-1 cells used in this study are used as a model of hematopoietic progenitor cells within the body. These cells, in both the laboratory setting as well as in the human body, can differentiate into promonocytic cells as well as T cells if exposed to specific sequences of cytokines. One mechanism by which HIV latency within these cell types is maintained is through the action of HDACs recruited by sequence-specific transcription factors to HIV-1. TSA is thought to activate HIV-1 transcription by inducing histone hyperacetylation within one of the regulatory nucleosomes, nuc-1, which is positioned immediately downstream from the transcription start site within the LTR. Acetylation of nuc-1 is considered to be a critical step in activation that precedes nuc-1 remodeling and, subsequently, transcriptional initiation. Within the stably transfected TF-1 and U-937 cells, TSA treatment alone did not result in an induction of LTR-driven gene transcription (data not shown). Within the LTRs containing the SNPs of interest in the TF-1 and U-937 cell clones, it could be thought that treatment of cell clones containing the 3T LAI LTR or 5T LAI LTRs with TSA may not result in an increase in LTR-driven gene expression due to the reduction of C/EBP and Sp transcription factors binding to their respective binding sites within those LTRs. However, the lack of induction of cell clones containing the WT LAI LTR is not explained by this phenomenon.

In contrast to the results obtained in the TF-1 and U-937 LAI LTR cell clones where TSA treatment did not result in increases in LTR-driven gene expression within many of the cell clones, treatment of stably transfected Jurkat clones containing the 3T and 5T LTRs had very different results. These results could potentially indicate that not only there is a SNP-specific response to TSA treatment, but also there is a cell-specific response. Jurkat cells, unlike the hematopoietic progenitor TF-1 cells or the promonocytic U-937 cells, are fully differentiated cells, meaning that they potentially contain higher levels of specific transcription factors within the nucleus, such as NF-*κ*B, necessary to enhance LTR-driven gene expression. Additionally, HDAC inhibitors, such as TSA, have anti-inflammatory properties and have been shown to reactivate latent HIV-1 virtually in the absence of T-cell activation, indicating that perhaps histone acetylation alone could affect reactivation of latent HIV-1 within T cells.

The TF-1, U-937, and Jurkat stably transfected cell clones were also treated with the combination of TSA and TNF-*α*, as previously described, to observe whether cell stimulation in conjunction with TSA treatment would enhance the effects of TSA, and possibly induce LTRs within the NE cells to drive higher levels of gene expression. While combination treatment resulted in induction of most of the IE and HE TF-1 cell clones, NE TF-1 cells containing the WT, 3T, or 5T LAI LTRs were not able to express higher levels of GFP. However, combination treatment did not increase induction of one of the IE clones containing the 3T LAI LTR, as well as a high GFP-expressing clone containing the 5T LAI LTR. This potentially indicates that HDACs alone may not be preventing HIV-1 LTR driven gene transcription within these cells (Figure 4(a)). Additionally, combination treatment in the U-937 NE clones did not lead to induction in gene transcription; however, the same treatment in IE and HE cells did result in an increase in LTR-driven GFP expression (Figure 4(b)). Again, in one IE clone containing the 3T LAI LTR and one HE clone containing the 5T LAI LTR, combination treatment only minimally induced LTR-driven gene expression again indicating that perhaps there is some other epigenetic control, perhaps cytosine methylation, leading to the repression in LTR-driven gene expression.

The largest changes in LTR-driven gene expression were observed in the NE Jurkat clone containing the 5T LAI LTR (Figure 5(d)). As stated earlier, the 5T SNP has been shown to result in a decrease in binding of the Sp transcription factors to the Sp binding site III. However, the LTR contains two other Sp binding sites that bind the Sp factors shown to recruit HATs to the LTR. Perhaps the other binding sites or the steric availability of the NF-*κ*B binding site, whose cognate transcription factors have also been shown to interact with both HDACs and HATs, led to interactions of the Sp factors or NF-*κ*B heterodimer with HATs due to the presence of the HDAC inhibitor and TNF-*α*.

To determine if the inhibitory effects of the HDACs could possibly be because of a specific HDAC, a commonly used HDAC inhibitor, valproic acid (VPA) against HDAC 1, was used in a range of concentrations utilized in other studies, to see if inhibition of HDAC 1 could lead to increases in LTR-driven gene transcription within these cells. Clones from all three cell lines across all four LAI LTRs that were used in this study were treated with and without VPA; however, there was no change in LTR-driven gene expression within any of the NE, IE, or HE cell clones (data not shown). Potentially, this result was due to the fact that there is more than just one histone regulator affecting LTR-driven gene expression and this is better captured through the data that was obtained using the general HDAC inhibitor TSA. VPA has been described to reactivate latent virus within the CD4^+^ T-cell reservoir without stimulation of the cells within four patients; however, in two separate subsequent studies, this finding was refuted, possibly due to the ineffectiveness of VPA to inhibit another HDAC activity in CD4+ T cells.

## 5. Conclusions

Pre- and post-HAART era viruses containing the 3T, 5T, and 3T5T genotypic variants within the LTR could potentially be involved in evading the immune system by altering the course of viral gene expression during HIV-1 infection of progenitor cells, which will subsequently have daughter cells carrying the integrated provirus that can produce more viruses to infect other cells as well as travel through the blood and cross the BBB to infect susceptible cells in end-organs such as the brain. This work demonstrates that the 3T-, 5T-, and 3T5T-containing LTRs induce different patterns of gene expression within TF-1, U-937, and Jurkat cells and these studies have indicated that there is SNP-specific as well as cell type-specific mechanism of action within these cells that lead to alterations in LTR-driven gene expression. These studies will enhance our understanding of HIV-1 disease and how it progresses within infected individuals as the virus adapts in response to selective pressures within the host during the course of disease.

Based on the outcome of these investigations, additional studies are warranted that will involve the use of primary cells and replication competent infectious molecular clones carrying LTR binding site variants of interest (3T, 5T, 3T5T, and perhaps others) in viral replication studies in vitro and perhaps in humanized mice. Gene expression within primary cells would potentially be different and could affect the presence or absence of specific transcription factors necessary for LTR function within specific cell types or physiologic conditions. In addition, all of these studies were conducted with cells that were stably transfected with a plasmid containing the HIV-1 LTR. It will be important to further define the phenotype of the variant C/EBP and Sp binding sites within the context of infectious molecular clones of selected viral genotypes/phenotypes.

As this work was performed in only one HIV-1 background (LAI), it will be critical to determine if genotypic differences within the LTRs of other HIV-1 backbones, such as YU-2 (R5-tropic) and 89.6 (R5/X4 dual tropic), would lead to similar results as observed with the LAI LTR backbone, or if the phenotype observed could be altered based on the differences in the backbone genotype. Studying the HIV-1 binding site variants within the context of the LTR and the whole viral genome would also open the door to continued exploration of the potential impact of other viral proteins such as Env, Nef, and Vpr on the replication properties of virus containing the C/EBP and Sp binding site variants of interest. In particular, it will be of interest to continue to explore the interaction of Vpr with the cis-acting elements spanning C/EBP site I, the NF-*κ*B core/enhancer region, and Sp site III. This experimental directive is based in part on previous studies that have demonstrated the specific interaction of Vpr with specific sequences contained within C/EBP binding site I and NF-*κ*B binding site II. Additionally, it will also be important to determine if the changes in LTR-driven gene expression within each of the cell types and each of the LTRs containing SNPs of interest are due to epigenetic factors that have been shown to control host as well as integrated HIV-1 gene expression.

This work demonstrates the effects of epigenetic regulators on LTR-driven gene expression within cells of the hematopoietic progenitor, promonocytic, and T-cell lines containing SNP-specific LTRs of interest. The results of this study reveal that genetic variation within Sp binding site III and C/EBP binding site I plays important roles in histone acetylation and deacetylation as demonstrated by the effects of TSA and TSA in combination with TNF-*α*. In particular, this work potentially demonstrates that T cells infected with virus containing the 5T variant can remain latent without any stimulation; however, with epigenetic modulators they can be reactivated to replicate and potentially generate high levels of virus. This work may aid in future development of therapies that can target reactivation of latent or quiescent viral quasispecies that have formed niches within hematopoietic progenitor, monocytic, and T cells, as well as the further development of paradigms to predict gene expression and latency phenotypes of HIV-1 isolates, and help to draw correlations between LTR genotype and viral replication within specific cell types.
